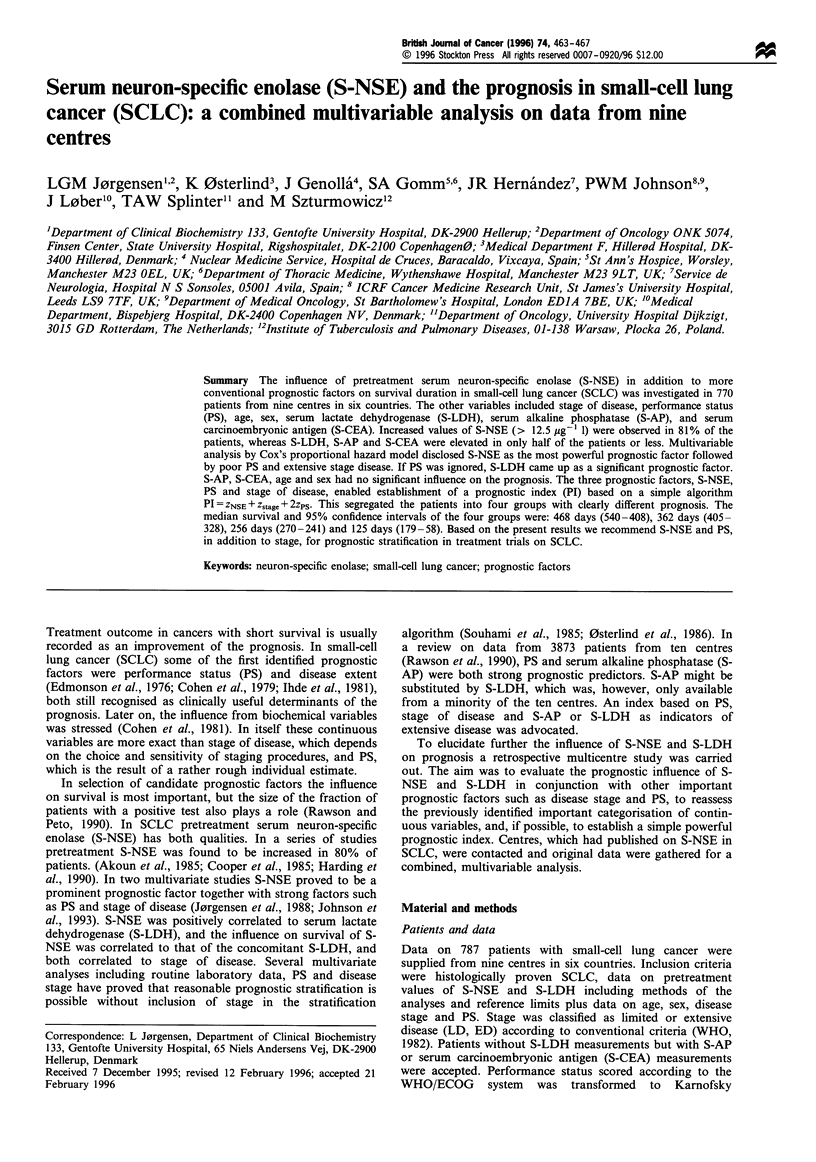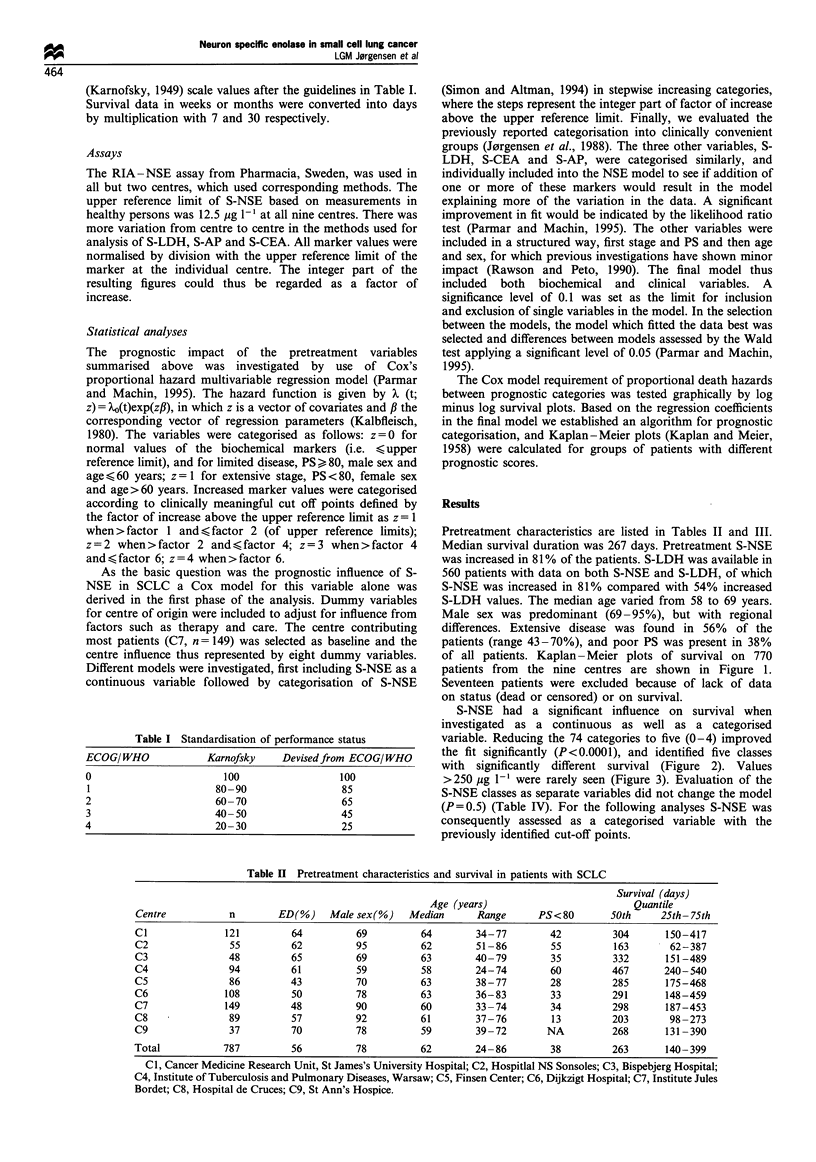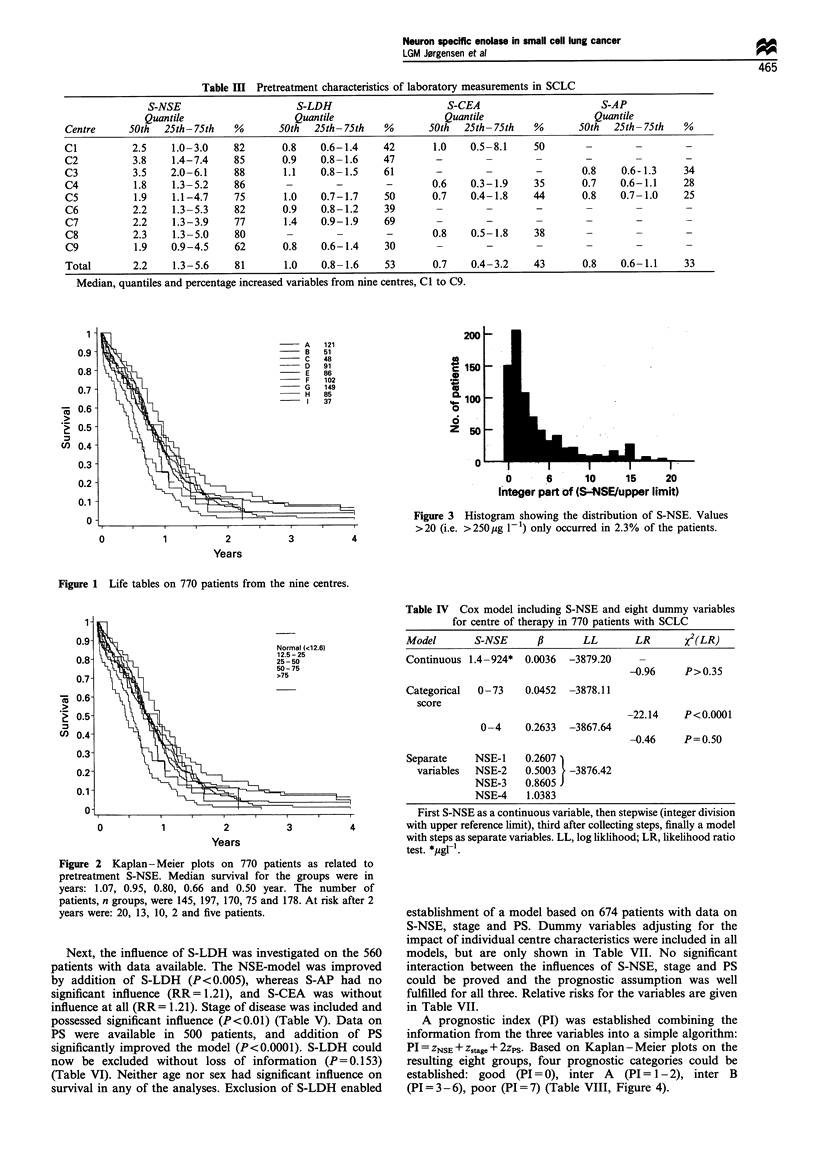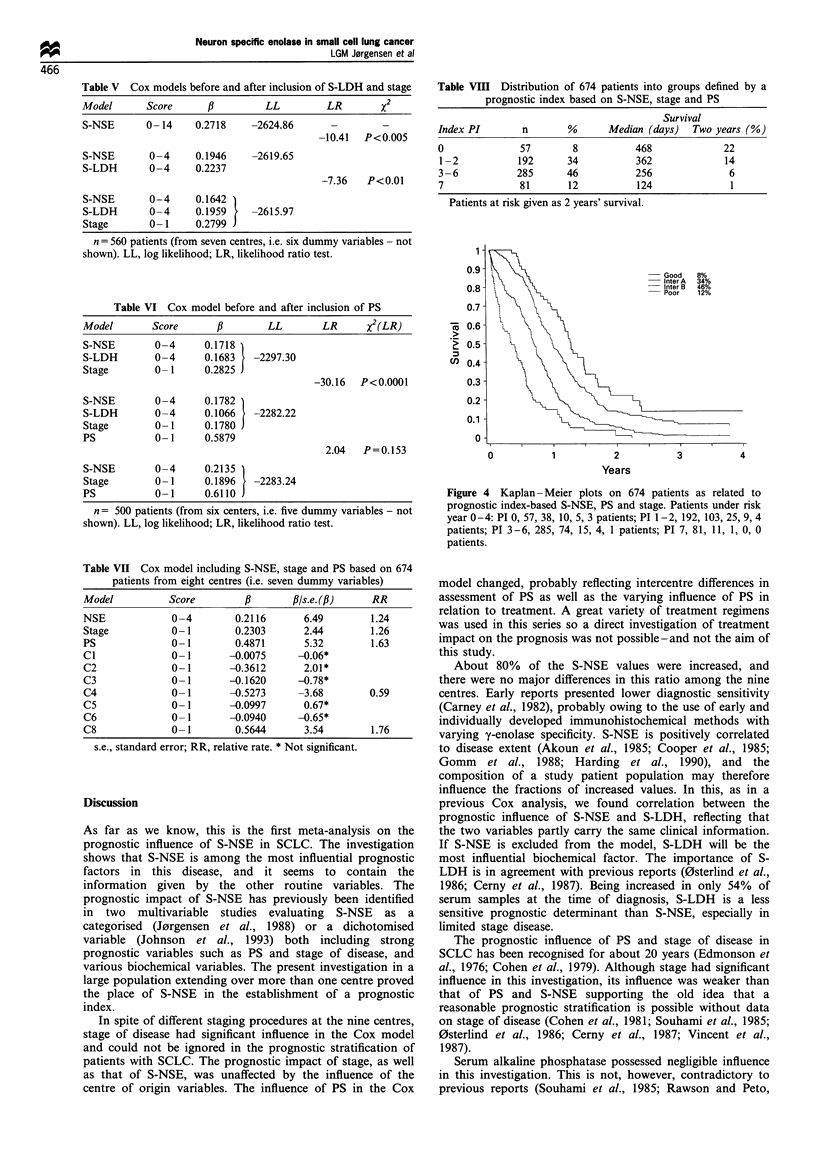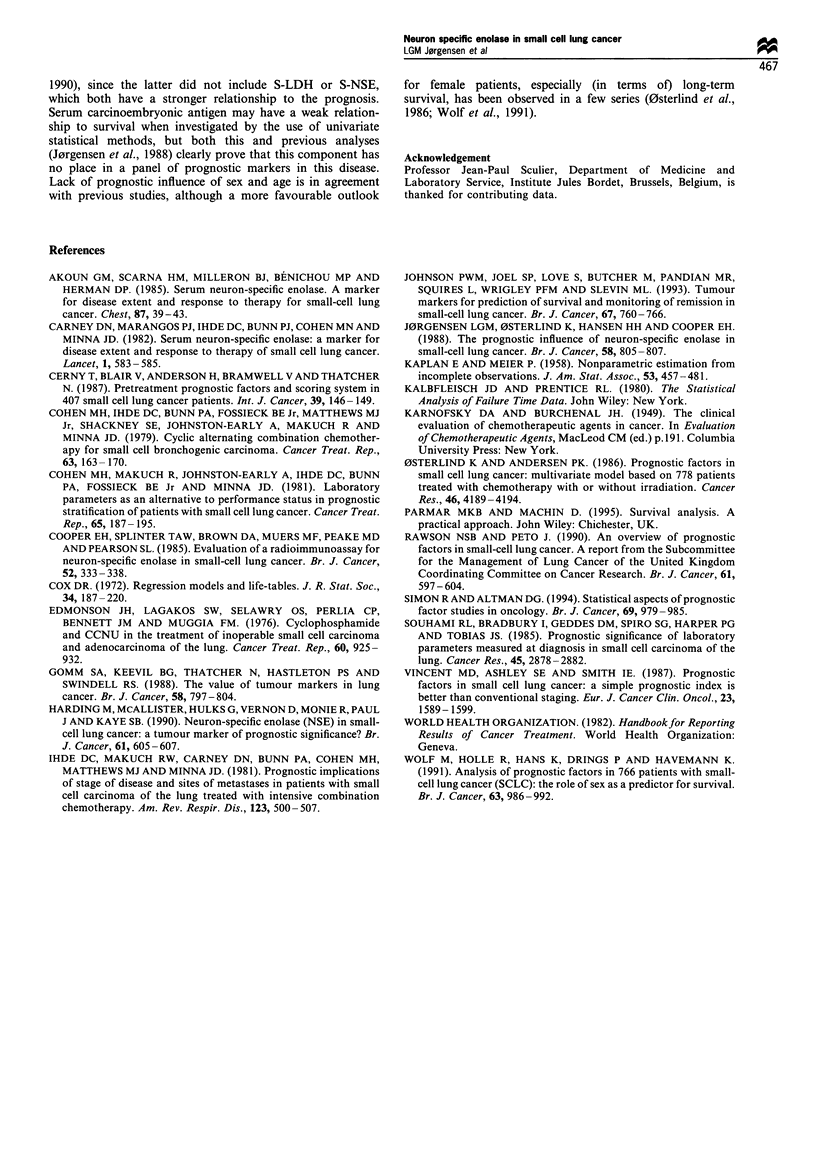# Serum neuron-specific enolase (S-NSE) and the prognosis in small-cell lung cancer (SCLC): a combined multivariable analysis on data from nine centres.

**DOI:** 10.1038/bjc.1996.383

**Published:** 1996-08

**Authors:** L. G. Jørgensen, K. Osterlind, J. Genollá, S. A. Gomm, J. R. Hernández, P. W. Johnson, J. Løber, T. A. Splinter, M. Szturmowicz

**Affiliations:** Department of Clinical Biochemistry 133, Gentofte University Hospital, Hellerup, Denmark.

## Abstract

The influence of pretreatment serum neuron-specific enolase (S-NSE) in addition to more conventional prognostic factors on survival duration in small-cell lung cancer (SCLC) was investigated in 770 patients from nine centres in six countries. The other variables included stage of disease, performance status (PS), age, sex, serum lactate dehydrogenase (S-LDH), serum alkaline phosphatase (S-AP), and serum carcinoembryonic antigen (S-CEA). Increased values of S-NSE (> 12.5 micrograms-1 l) were observed in 81% of the patients, whereas S-LDH, S-AP and S-CEA were elevated in only half of the patients or less. Multivariable analysis by Cox's proportional hazard model disclosed S-NSE as the most powerful prognostic factor followed by poor PS and extensive stage disease. If PS was ignored, S-LDH came up as a significant prognostic factor. S-AP, S-CEA, age and sex had no significant influence on the prognosis. The three prognostic factors, S-NSE, PS and stage of disease, enabled establishment of a prognostic index (PI) based on a simple algorithm PI = zNSE + z(stage) + 2zPS. This segregated the patients into four groups with clearly different prognosis. The median survival and 95% confidence intervals of the four groups were: 468 days (540-408), 362 days (405-328), 256 days (270-241) and 125 days (179-58). Based on the present results we recommend S-NSE and PS, in addition to stage, for prognostic stratification in treatment trials on SCLC.


British Journal of Cancer (1996) 74, 463-467
© 1996 Stockton Press All rights reserved 0007-0920/96 $12.00

### Serum neuron-specific enolase (S-NSE) and the prognosis in small-cell lung cancer (SCLC): a combined multivariable analysis on data from nine centres

LGM Jørgensen[1,2], K Østerlind[3], J Genollá[4], SA Gomm[5,6], JR Hernández[7], PWM Johnson[8,9], J Løber[10], TAW Splinter[11] and M Szturmowicz[12]

*[1]Department of Clinical Biochemistry 133, Gentofte University Hospital, DK-2900 Hellerup; [2]Department of Oncology ONK 5074, Finsen Center, State University Hospital, Rigshospitalet, DK-2100 CopenhagenØ; [3]Medical Department F, Hillerød Hospital, DK-3400 Hillerød, Denmark; [4] Nuclear Medicine Service, Hospital de Cruces, Baracaldo, Vixcaya, Spain; [5]St Ann's Hospice, Worsley, Manchester M23 0EL, UK; [6]Department of Thoracic Medicine, Wythenshawe Hospital, Manchester M23 9LT, UK; [7]Service de Neurologia, Hospital N S Sonsoles, 05001 Avila, Spain; [8] ICRF Cancer Medicine Research Unit, St James's University Hospital, Leeds LS9 7TF, UK; [9]Department of Medical Oncology, St Bartholomew's Hospital, London ED1A 7BE, UK; [10]Medical Department, Bispebjerg Hospital, DK-2400 Copenhagen NV, Denmark; [11]Department of Oncology, University Hospital Dijkzigt, 3015 GD Rotterdam, The Netherlands; [12]Institute of Tuberculosis and Pulmonary Diseases, 01-138 Warsaw, Plocka 26, Poland.*

**Summary** The influence of pretreatment serum neuron-specific enolase (S-NSE) in addition to more conventional prognostic factors on survival duration in small-cell lung cancer (SCLC) was investigated in 770 patients from nine centres in six countries. The other variables included stage of disease, performance status (PS), age, sex, serum lactate dehydrogenase (S-LDH), serum alkaline phosphatase (S-AP), and serum carcinoembryonic antigen (S-CEA). Increased values of S-NSE (> 12.5 $\mu g^{-1}$ l) were observed in 81% of the patients, whereas S-LDH, S-AP and S-CEA were elevated in only half of the patients or less. Multivariable analysis by Cox's proportional hazard model disclosed S-NSE as the most powerful prognostic factor followed by poor PS and extensive stage disease. If PS was ignored, S-LDH came up as a significant prognostic factor. S-AP, S-CEA, age and sex had no significant influence on the prognosis. The three prognostic factors, S-NSE, PS and stage of disease, enabled establishment of a prognostic index (PI) based on a simple algorithm $PI = z_{NSE} + z_{stage} + 2z_{PS}$. This segregated the patients into four groups with clearly different prognosis. The median survival and 95% confidence intervals of the four groups were: 468 days (540-408), 362 days (405-328), 256 days (270-241) and 125 days (179-58). Based on the present results we recommend S-NSE and PS, in addition to stage, for prognostic stratification in treatment trials on SCLC.

**Keywords:** neuron-specific enolase; small-cell lung cancer; prognostic factors

Treatment outcome in cancers with short survival is usually recorded as an improvement of the prognosis. In small-cell lung cancer (SCLC) some of the first identified prognostic factors were performance status (PS) and disease extent (Edmonson *et al.*, 1976; Cohen *et al.*, 1979; Ihde *et al.*, 1981), both still recognised as clinically useful determinants of the prognosis. Later on, the influence from biochemical variables was stressed (Cohen *et al.*, 1981). In itself these continuous variables are more exact than stage of disease, which depends on the choice and sensitivity of staging procedures, and PS, which is the result of a rather rough individual estimate.

In selection of candidate prognostic factors the influence on survival is most important, but the size of the fraction of patients with a positive test also plays a role (Rawson and Peto, 1990). In SCLC pretreatment serum neuron-specific enolase (S-NSE) has both qualities. In a series of studies pretreatment S-NSE was found to be increased in 80% of patients. (Akoun *et al.*, 1985; Cooper *et al.*, 1985; Harding *et al.*, 1990). In two multivariate studies S-NSE proved to be a prominent prognostic factor together with strong factors such as PS and stage of disease (Jørgensen *et al.*, 1988; Johnson *et al.*, 1993). S-NSE was positively correlated to serum lactate dehydrogenase (S-LDH), and the influence on survival of S-NSE was correlated to that of the concomitant S-LDH, and both correlated to stage of disease. Several multivariate analyses including routine laboratory data, PS and disease stage have proved that reasonable prognostic stratification is possible without inclusion of stage in the stratification algorithm (Souhami *et al.*, 1985; Østerlind *et al.*, 1986). In a review on data from 3873 patients from ten centres (Rawson *et al.*, 1990), PS and serum alkaline phosphatase (S-AP) were both strong prognostic predictors. S-AP might be substituted by S-LDH, which was, however, only available from a minority of the ten centres. An index based on PS, stage of disease and S-AP or S-LDH as indicators of extensive disease was advocated.

To elucidate further the influence of S-NSE and S-LDH on prognosis a retrospective multicentre study was carried out. The aim was to evaluate the prognostic influence of S-NSE and S-LDH in conjunction with other important prognostic factors such as disease stage and PS, to reassess the previously identified important categorisation of continuous variables, and, if possible, to establish a simple powerful prognostic index. Centres, which had published on S-NSE in SCLC, were contacted and original data were gathered for a combined, multivariable analysis.

### Material and methods

### *Patients and data*

Data on 787 patients with small-cell lung cancer were supplied from nine centres in six countries. Inclusion criteria were histologically proven SCLC, data on pretreatment values of S-NSE and S-LDH including methods of the analyses and reference limits plus data on age, sex, disease stage and PS. Stage was classified as limited or extensive disease (LD, ED) according to conventional criteria (WHO, 1982). Patients without S-LDH measurements but with S-AP or serum carcinoembryonic antigen (S-CEA) measurements were accepted. Performance status scored according to the WHO/ECOG system was transformed to Karnofsky

Correspondence: L Jørgensen, Department of Clinical Biochemistry 133, Gentofte University Hospital, 65 Niels Andersens Vej, DK-2900 Hellerup, Denmark
Received 7 December 1995; revised 12 February 1996; accepted 21 February 1996

(Karnofsky, 1949) scale values after the guidelines in Table I. Survival data in weeks or months were converted into days by multiplication with 7 and 30 respectively.

*Assays*

The RIA–NSE assay from Pharmacia, Sweden, was used in all but two centres, which used corresponding methods. The upper reference limit of S-NSE based on measurements in healthy persons was 12.5 $\mu g\ l^{-1}$ at all nine centres. There was more variation from centre to centre in the methods used for analysis of S-LDH, S-AP and S-CEA. All marker values were normalised by division with the upper reference limit of the marker at the individual centre. The integer part of the resulting figures could thus be regarded as a factor of increase.

*Statistical analyses*

The prognostic impact of the pretreatment variables summarised above was investigated by use of Cox's proportional hazard multivariable regression model (Parmar and Machin, 1995). The hazard function is given by $\lambda$ (t; $z) = \lambda_0(t)\exp(z\beta)$, in which $z$ is a vector of covariates and $\beta$ the corresponding vector of regression parameters (Kalbfleisch, 1980). The variables were categorised as follows: $z=0$ for normal values of the biochemical markers (i.e. $\leqslant$upper reference limit), and for limited disease, PS$\geqslant$80, male sex and age$\leqslant$60 years; $z=1$ for extensive stage, PS<80, female sex and age>60 years. Increased marker values were categorised according to clinically meaningful cut off points defined by the factor of increase above the upper reference limit as $z=1$ when>factor 1 and$\leqslant$factor 2 (of upper reference limits); $z=2$ when>factor 2 and$\leqslant$factor 4; $z=3$ when>factor 4 and$\leqslant$factor 6; $z=4$ when>factor 6.

As the basic question was the prognostic influence of S-NSE in SCLC a Cox model for this variable alone was derived in the first phase of the analysis. Dummy variables for centre of origin were included to adjust for influence from factors such as therapy and care. The centre contributing most patients (C7, $n=149$) was selected as baseline and the centre influence thus represented by eight dummy variables. Different models were investigated, first including S-NSE as a continuous variable followed by categorisation of S-NSE (Simon and Altman, 1994) in stepwise increasing categories, where the steps represent the integer part of factor of increase above the upper reference limit. Finally, we evaluated the previously reported categorisation into clinically convenient groups (Jørgensen *et al*., 1988). The three other variables, S-LDH, S-CEA and S-AP, were categorised similarly, and individually included into the NSE model to see if addition of one or more of these markers would result in the model explaining more of the variation in the data. A significant improvement in fit would be indicated by the likelihood ratio test (Parmar and Machin, 1995). The other variables were included in a structured way, first stage and PS and then age and sex, for which previous investigations have shown minor impact (Rawson and Peto, 1990). The final model thus included both biochemical and clinical variables. A significance level of 0.1 was set as the limit for inclusion and exclusion of single variables in the model. In the selection between the models, the model which fitted the data best was selected and differences between models assessed by the Wald test applying a significant level of 0.05 (Parmar and Machin, 1995).

The Cox model requirement of proportional death hazards between prognostic categories was tested graphically by log minus log survival plots. Based on the regression coefficients in the final model we established an algorithm for prognostic categorisation, and Kaplan–Meier plots (Kaplan and Meier, 1958) were calculated for groups of patients with different prognostic scores.

**Table I** Standardisation of performance status

| *ECOG/WHO* | *Karnofsky* | *Devised from ECOG/WHO* |
|---|---|---|
| 0 | 100 | 100 |
| 1 | 80–90 | 85 |
| 2 | 60–70 | 65 |
| 3 | 40–50 | 45 |
| 4 | 20–30 | 25 |

### Results

Pretreatment characteristics are listed in Tables II and III. Median survival duration was 267 days. Pretreatment S-NSE was increased in 81% of the patients. S-LDH was available in 560 patients with data on both S-NSE and S-LDH, of which S-NSE was increased in 81% compared with 54% increased S-LDH values. The median age varied from 58 to 69 years. Male sex was predominant (69–95%), but with regional differences. Extensive disease was found in 56% of the patients (range 43–70%), and poor PS was present in 38% of all patients. Kaplan–Meier plots of survival on 770 patients from the nine centres are shown in Figure 1. Seventeen patients were excluded because of lack of data on status (dead or censored) or on survival.

S-NSE had a significant influence on survival when investigated as a continuous as well as a categorised variable. Reducing the 74 categories to five (0–4) improved the fit significantly ($P<0.0001$), and identified five classes with significantly different survival (Figure 2). Values >250 $\mu g\ l^{-1}$ were rarely seen (Figure 3). Evaluation of the S-NSE classes as separate variables did not change the model ($P=0.5$) (Table IV). For the following analyses S-NSE was consequently assessed as a categorised variable with the previously identified cut-off points.

**Table II** Pretreatment characteristics and survival in patients with SCLC

| | | | | *Age (years)* | | | *Survival (days) Quantile* | |
|---|---|---|---|---|---|---|---|---|
| *Centre* | n | *ED(%)* | *Male sex(%)* | *Median* | *Range* | *PS<80* | *50th* | *25th–75th* |
| C1 | 121 | 64 | 69 | 64 | 34–77 | 42 | 304 | 150–417 |
| C2 | 55 | 62 | 95 | 62 | 51–86 | 55 | 163 | 62–387 |
| C3 | 48 | 65 | 69 | 63 | 40–79 | 35 | 332 | 151–489 |
| C4 | 94 | 61 | 59 | 58 | 24–74 | 60 | 467 | 240–540 |
| C5 | 86 | 43 | 70 | 63 | 38–77 | 28 | 285 | 175–468 |
| C6 | 108 | 50 | 78 | 63 | 36–83 | 33 | 291 | 148–459 |
| C7 | 149 | 48 | 90 | 60 | 33–74 | 34 | 298 | 187–453 |
| C8 | 89 | 57 | 92 | 61 | 37–76 | 13 | 203 | 98–273 |
| C9 | 37 | 70 | 78 | 59 | 39–72 | NA | 268 | 131–390 |
| Total | 787 | 56 | 78 | 62 | 24–86 | 38 | 263 | 140–399 |

C1, Cancer Medicine Research Unit, St James's University Hospital; C2, Hospitlal NS Sonsoles; C3, Bispebjerg Hospital; C4, Institute of Tuberculosis and Pulmonary Diseases, Warsaw; C5, Finsen Center; C6, Dijkzigt Hospital; C7, Institute Jules Bordet; C8, Hospital de Cruces; C9, St Ann's Hospice.

**Table III** Pretreatment characteristics of laboratory measurements in SCLC

| Centre | *S-NSE Quantile* 50th | 25th–75th | % | *S-LDH Quantile* 50th | 25th–75th | % | *S-CEA Quantile* 50th | 25th–75th | % | *S-AP Quantile* 50th | 25th–75th | % |
|---|---|---|---|---|---|---|---|---|---|---|---|---|
| C1 | 2.5 | 1.0–3.0 | 82 | 0.8 | 0.6–1.4 | 42 | 1.0 | 0.5–8.1 | 50 | – | – | – |
| C2 | 3.8 | 1.4–7.4 | 85 | 0.9 | 0.8–1.6 | 47 | – | – | – | – | – | – |
| C3 | 3.5 | 2.0–6.1 | 88 | 1.1 | 0.8–1.5 | 61 | – | – | – | 0.8 | 0.6–1.3 | 34 |
| C4 | 1.8 | 1.3–5.2 | 86 | – | – | – | 0.6 | 0.3–1.9 | 35 | 0.7 | 0.6–1.1 | 28 |
| C5 | 1.9 | 1.1–4.7 | 75 | 1.0 | 0.7–1.7 | 50 | 0.7 | 0.4–1.8 | 44 | 0.8 | 0.7–1.0 | 25 |
| C6 | 2.2 | 1.3–5.3 | 82 | 0.9 | 0.8–1.2 | 39 | – | – | – | – | – | – |
| C7 | 2.2 | 1.3–3.9 | 77 | 1.4 | 0.9–1.9 | 69 | – | – | – | – | – | – |
| C8 | 2.3 | 1.3–5.0 | 80 | – | – | – | 0.8 | 0.5–1.8 | 38 | – | – | – |
| C9 | 1.9 | 0.9–4.5 | 62 | 0.8 | 0.6–1.4 | 30 | – | – | – | – | – | – |
| Total | 2.2 | 1.3–5.6 | 81 | 1.0 | 0.8–1.6 | 53 | 0.7 | 0.4–3.2 | 43 | 0.8 | 0.6–1.1 | 33 |

Median, quantiles and percentage increased variables from nine centres, C1 to C9.

**Figure 1** Life tables on 770 patients from the nine centres.

**Figure 2** Kaplan–Meier plots on 770 patients as related to pretreatment S-NSE. Median survival for the groups were in years: 1.07, 0.95, 0.80, 0.66 and 0.50 year. The number of patients, *n* groups, were 145, 197, 170, 75 and 178. At risk after 2 years were: 20, 13, 10, 2 and five patients.

**Figure 3** Histogram showing the distribution of S-NSE. Values >20 (i.e. >250 $\mu g\ l^{-1}$) only occurred in 2.3% of the patients.

**Table IV** Cox model including S-NSE and eight dummy variables for centre of therapy in 770 patients with SCLC

| *Model* | *S-NSE* | *β* | *LL* | *LR* | *$\chi^2$(LR)* |
|---|---|---|---|---|---|
| Continuous | 1.4–924* | 0.0036 | –3879.20 | – | |
| | | | | –0.96 | $P>0.35$ |
| Categorical score | 0–73 | 0.0452 | –3878.11 | | |
| | | | | –22.14 | $P<0.0001$ |
| | 0–4 | 0.2633 | –3867.64 | | |
| | | | | –0.46 | $P=0.50$ |
| Separate variables | NSE-1 | 0.2607 | –3876.42 | | |
| | NSE-2 | 0.5003 | | | |
| | NSE-3 | 0.8605 | | | |
| | NSE-4 | 1.0383 | | | |

First S-NSE as a continuous variable, then stepwise (integer division with upper reference limit), third after collecting steps, finally a model with steps as separate variables. LL, log liklihood; LR, likelihood ratio test. *$\mu g l^{-1}$.

Next, the influence of S-LDH was investigated on the 560 patients with data available. The NSE-model was improved by addition of S-LDH ($P<0.005$), whereas S-AP had no significant influence (RR = 1.21), and S-CEA was without influence at all (RR = 1.21). Stage of disease was included and possessed significant influence ($P<0.01$) (Table V). Data on PS were available in 500 patients, and addition of PS significantly improved the model ($P<0.0001$). S-LDH could now be excluded without loss of information ($P=0.153$) (Table VI). Neither age nor sex had significant influence on survival in any of the analyses. Exclusion of S-LDH enabled establishment of a model based on 674 patients with data on S-NSE, stage and PS. Dummy variables adjusting for the impact of individual centre characteristics were included in all models, but are only shown in Table VII. No significant interaction between the influences of S-NSE, stage and PS could be proved and the prognostic assumption was well fulfilled for all three. Relative risks for the variables are given in Table VII.

A prognostic index (PI) was established combining the information from the three variables into a simple algorithm: $PI = z_{NSE} + z_{stage} + 2z_{PS}$. Based on Kaplan–Meier plots on the resulting eight groups, four prognostic categories could be established: good (PI = 0), inter A (PI = 1–2), inter B (PI = 3–6), poor (PI = 7) (Table VIII, Figure 4).

**Table V** Cox models before and after inclusion of S-LDH and stage

| *Model* | *Score* | *β* | *LL* | *LR* | $\chi^2$ |
|---|---|---|---|---|---|
| S-NSE | 0-14 | 0.2718 | -2624.86 | – | – |
| | | | | -10.41 | $P<0.005$ |
| S-NSE | 0-4 | 0.1946 | -2619.65 | | |
| S-LDH | 0-4 | 0.2237 | | | |
| | | | | -7.36 | $P<0.01$ |
| S-NSE | 0-4 | 0.1642 | -2615.97 | | |
| S-LDH | 0-4 | 0.1959 | | | |
| Stage | 0-1 | 0.2799 | | | |

$n=560$ patients (from seven centres, i.e. six dummy variables – not shown). LL, log likelihood; LR, likelihood ratio test.

**Table VI** Cox model before and after inclusion of PS

| *Model* | *Score* | *β* | *LL* | *LR* | $\chi^2(LR)$ |
|---|---|---|---|---|---|
| S-NSE | 0-4 | 0.1718 | -2297.30 | | |
| S-LDH | 0-4 | 0.1683 | | | |
| Stage | 0-1 | 0.2825 | | | |
| | | | | -30.16 | $P<0.0001$ |
| S-NSE | 0-4 | 0.1782 | -2282.22 | | |
| S-LDH | 0-4 | 0.1066 | | | |
| Stage | 0-1 | 0.1780 | | | |
| PS | 0-1 | 0.5879 | | | |
| | | | | 2.04 | $P=0.153$ |
| S-NSE | 0-4 | 0.2135 | -2283.24 | | |
| Stage | 0-1 | 0.1896 | | | |
| PS | 0-1 | 0.6110 | | | |

$n=$ 500 patients (from six centers, i.e. five dummy variables – not shown). LL, log likelihood; LR, likelihood ratio test.

**Table VII** Cox model including S-NSE, stage and PS based on 674 patients from eight centres (i.e. seven dummy variables)

| *Model* | *Score* | *β* | *β/s.e.(β)* | *RR* |
|---|---|---|---|---|
| NSE | 0-4 | 0.2116 | 6.49 | 1.24 |
| Stage | 0-1 | 0.2303 | 2.44 | 1.26 |
| PS | 0-1 | 0.4871 | 5.32 | 1.63 |
| C1 | 0-1 | -0.0075 | -0.06* | |
| C2 | 0-1 | -0.3612 | 2.01* | |
| C3 | 0-1 | -0.1620 | -0.78* | |
| C4 | 0-1 | -0.5273 | -3.68 | 0.59 |
| C5 | 0-1 | -0.0997 | 0.67* | |
| C6 | 0-1 | -0.0940 | -0.65* | |
| C8 | 0-1 | 0.5644 | 3.54 | 1.76 |

s.e., standard error; RR, relative rate. * Not significant.

**Table VIII** Distribution of 674 patients into groups defined by a prognostic index based on S-NSE, stage and PS

| | | | *Survival* | |
|---|---|---|---|---|
| *Index PI* | n | % | *Median (days)* | *Two years (%)* |
| 0 | 57 | 8 | 468 | 22 |
| 1-2 | 192 | 34 | 362 | 14 |
| 3-6 | 285 | 46 | 256 | 6 |
| 7 | 81 | 12 | 124 | 1 |

Patients at risk given as 2 years' survival.

**Figure 4** Kaplan–Meier plots on 674 patients as related to prognostic index-based S-NSE, PS and stage. Patients under risk year 0–4: PI 0, 57, 38, 10, 5, 3 patients; PI 1–2, 192, 103, 25, 9, 4 patients; PI 3–6, 285, 74, 15, 4, 1 patients; PI 7, 81, 11, 1, 0, 0 patients.

### Discussion

As far as we know, this is the first meta-analysis on the prognostic influence of S-NSE in SCLC. The investigation shows that S-NSE is among the most influential prognostic factors in this disease, and it seems to contain the information given by the other routine variables. The prognostic impact of S-NSE has previously been identified in two multivariable studies evaluating S-NSE as a categorised (Jørgensen *et al.*, 1988) or a dichotomised variable (Johnson *et al.*, 1993) both including strong prognostic variables such as PS and stage of disease, and various biochemical variables. The present investigation in a large population extending over more than one centre proved the place of S-NSE in the establishment of a prognostic index.

In spite of different staging procedures at the nine centres, stage of disease had significant influence in the Cox model and could not be ignored in the prognostic stratification of patients with SCLC. The prognostic impact of stage, as well as that of S-NSE, was unaffected by the influence of the centre of origin variables. The influence of PS in the Cox model changed, probably reflecting intercentre differences in assessment of PS as well as the varying influence of PS in relation to treatment. A great variety of treatment regimens was used in this series so a direct investigation of treatment impact on the prognosis was not possible–and not the aim of this study.

About 80% of the S-NSE values were increased, and there were no major differences in this ratio among the nine centres. Early reports presented lower diagnostic sensitivity (Carney *et al.*, 1982), probably owing to the use of early and individually developed immunohistochemical methods with varying γ-enolase specificity. S-NSE is positively correlated to disease extent (Akoun *et al.*, 1985; Cooper *et al.*, 1985; Gomm *et al.*, 1988; Harding *et al.*, 1990), and the composition of a study patient population may therefore influence the fractions of increased values. In this, as in a previous Cox analysis, we found correlation between the prognostic influence of S-NSE and S-LDH, reflecting that the two variables partly carry the same clinical information. If S-NSE is excluded from the model, S-LDH will be the most influential biochemical factor. The importance of S-LDH is in agreement with previous reports (Østerlind *et al.*, 1986; Cerny *et al.*, 1987). Being increased in only 54% of serum samples at the time of diagnosis, S-LDH is a less sensitive prognostic determinant than S-NSE, especially in limited stage disease.

The prognostic influence of PS and stage of disease in SCLC has been recognised for about 20 years (Edmonson *et al.*, 1976; Cohen *et al.*, 1979). Although stage had significant influence in this investigation, its influence was weaker than that of PS and S-NSE supporting the old idea that a reasonable prognostic stratification is possible without data on stage of disease (Cohen *et al.*, 1981; Souhami *et al.*, 1985; Østerlind *et al.*, 1986; Cerny *et al.*, 1987; Vincent *et al.*, 1987).

Serum alkaline phosphatase possessed negligible influence in this investigation. This is not, however, contradictory to previous reports (Souhami *et al.*, 1985; Rawson and Peto,

1990), since the latter did not include S-LDH or S-NSE, which both have a stronger relationship to the prognosis. Serum carcinoembryonic antigen may have a weak relationship to survival when investigated by the use of univariate statistical methods, but both this and previous analyses (Jørgensen *et al.*, 1988) clearly prove that this component has no place in a panel of prognostic markers in this disease. Lack of prognostic influence of sex and age is in agreement with previous studies, although a more favourable outlook for female patients, especially (in terms of) long-term survival, has been observed in a few series (Østerlind *et al.*, 1986; Wolf *et al.*, 1991).

**Acknowledgement**
Professor Jean-Paul Sculier, Department of Medicine and Laboratory Service, Institute Jules Bordet, Brussels, Belgium, is thanked for contributing data.

### References

AKOUN GM, SCARNA HM, MILLERON BJ, BÉNICHOU MP AND HERMAN DP. (1985). Serum neuron-specific enolase. A marker for disease extent and response to therapy for small-cell lung cancer. *Chest*, **87**, 39–43.

CARNEY DN, MARANGOS PJ, IHDE DC, BUNN PJ, COHEN MN AND MINNA JD. (1982). Serum neuron-specific enolase: a marker for disease extent and response to therapy of small cell lung cancer. *Lancet*, **1**, 583–585.

CERNY T, BLAIR V, ANDERSON H, BRAMWELL V AND THATCHER N. (1987). Pretreatment prognostic factors and scoring system in 407 small cell lung cancer patients. *Int. J. Cancer*, **39**, 146–149.

COHEN MH, IHDE DC, BUNN PA, FOSSIECK BE Jr, MATTHEWS MJ Jr, SHACKNEY SE, JOHNSTON-EARLY A, MAKUCH R AND MINNA JD. (1979). Cyclic alternating combination chemotherapy for small cell bronchogenic carcinoma. *Cancer Treat. Rep.*, **63**, 163–170.

COHEN MH, MAKUCH R, JOHNSTON-EARLY A, IHDE DC, BUNN PA, FOSSIECK BE Jr AND MINNA JD. (1981). Laboratory parameters as an alternative to performance status in prognostic stratification of patients with small cell lung cancer. *Cancer Treat. Rep.*, **65**, 187–195.

COOPER EH, SPLINTER TAW, BROWN DA, MUERS MF, PEAKE MD AND PEARSON SL. (1985). Evaluation of a radioimmunoassay for neuron-specific enolase in small-cell lung cancer. *Br. J. Cancer*, **52**, 333–338.

COX DR. (1972). Regression models and life-tables. *J. R. Stat. Soc.*, **34**, 187–220.

EDMONSON JH, LAGAKOS SW, SELAWRY OS, PERLIA CP, BENNETT JM AND MUGGIA FM. (1976). Cyclophosphamide and CCNU in the treatment of inoperable small cell carcinoma and adenocarcinoma of the lung. *Cancer Treat. Rep.*, **60**, 925–932.

GOMM SA, KEEVIL BG, THATCHER N, HASTLETON PS AND SWINDELL RS. (1988). The value of tumour markers in lung cancer. *Br. J. Cancer*, **58**, 797–804.

HARDING M, MCALLISTER, HULKS G, VERNON D, MONIE R, PAUL J AND KAYE SB. (1990). Neuron-specific enolase (NSE) in small-cell lung cancer: a tumour marker of prognostic significance? *Br. J. Cancer*, **61**, 605–607.

IHDE DC, MAKUCH RW, CARNEY DN, BUNN PA, COHEN MH, MATTHEWS MJ AND MINNA JD. (1981). Prognostic implications of stage of disease and sites of metastases in patients with small cell carcinoma of the lung treated with intensive combination chemotherapy. *Am. Rev. Respir. Dis.*, **123**, 500–507.

JOHNSON PWM, JOEL SP, LOVE S, BUTCHER M, PANDIAN MR, SQUIRES L, WRIGLEY PFM AND SLEVIN ML. (1993). Tumour markers for prediction of survival and monitoring of remission in small-cell lung cancer. *Br. J. Cancer*, **67**, 760–766.

JØRGENSEN LGM, ØSTERLIND K, HANSEN HH AND COOPER EH. (1988). The prognostic influence of neuron-specific enolase in small-cell lung cancer. *Br. J. Cancer*, **58**, 805–807.

KAPLAN E AND MEIER P. (1958). Nonparametric estimation from incomplete observations. *J. Am. Stat. Assoc.*, **53**, 457–481.

KALBFLEISCH JD AND PRENTICE RL. (1980). *The Statistical Analysis of Failure Time Data*. John Wiley: New York.

KARNOFSKY DA AND BURCHENAL JH. (1949). The clinical evaluation of chemotherapeutic agents in cancer. In *Evaluation of Chemotherapeutic Agents*, MacLeod CM (ed.) p.191. Columbia University Press: New York.

ØSTERLIND K AND ANDERSEN PK. (1986). Prognostic factors in small cell lung cancer: multivariate model based on 778 patients treated with chemotherapy with or without irradiation. *Cancer Res.*, **46**, 4189–4194.

PARMAR MKB AND MACHIN D. (1995). Survival analysis. A practical approach. John Wiley: Chichester, UK.

RAWSON NSB AND PETO J. (1990). An overview of prognostic factors in small-cell lung cancer. A report from the Subcommittee for the Management of Lung Cancer of the United Kingdom Coordinating Committee on Cancer Research. *Br. J. Cancer*, **61**, 597–604.

SIMON R AND ALTMAN DG. (1994). Statistical aspects of prognostic factor studies in oncology. *Br. J. Cancer*, **69**, 979–985.

SOUHAMI RL, BRADBURY I, GEDDES DM, SPIRO SG, HARPER PG AND TOBIAS JS. (1985). Prognostic significance of laboratory parameters measured at diagnosis in small cell carcinoma of the lung. *Cancer Res.*, **45**, 2878–2882.

VINCENT MD, ASHLEY SE AND SMITH IE. (1987). Prognostic factors in small cell lung cancer: a simple prognostic index is better than conventional staging. *Eur. J. Cancer Clin. Oncol.*, **23**, 1589–1599.

WORLD HEALTH ORGANIZATION. (1982). *Handbook for Reporting Results of Cancer Treatment*. World Health Organization: Geneva.

WOLF M, HOLLE R, HANS K, DRINGS P AND HAVEMANN K. (1991). Analysis of prognostic factors in 766 patients with small-cell lung cancer (SCLC): the role of sex as a predictor for survival. *Br. J. Cancer*, **63**, 986–992.