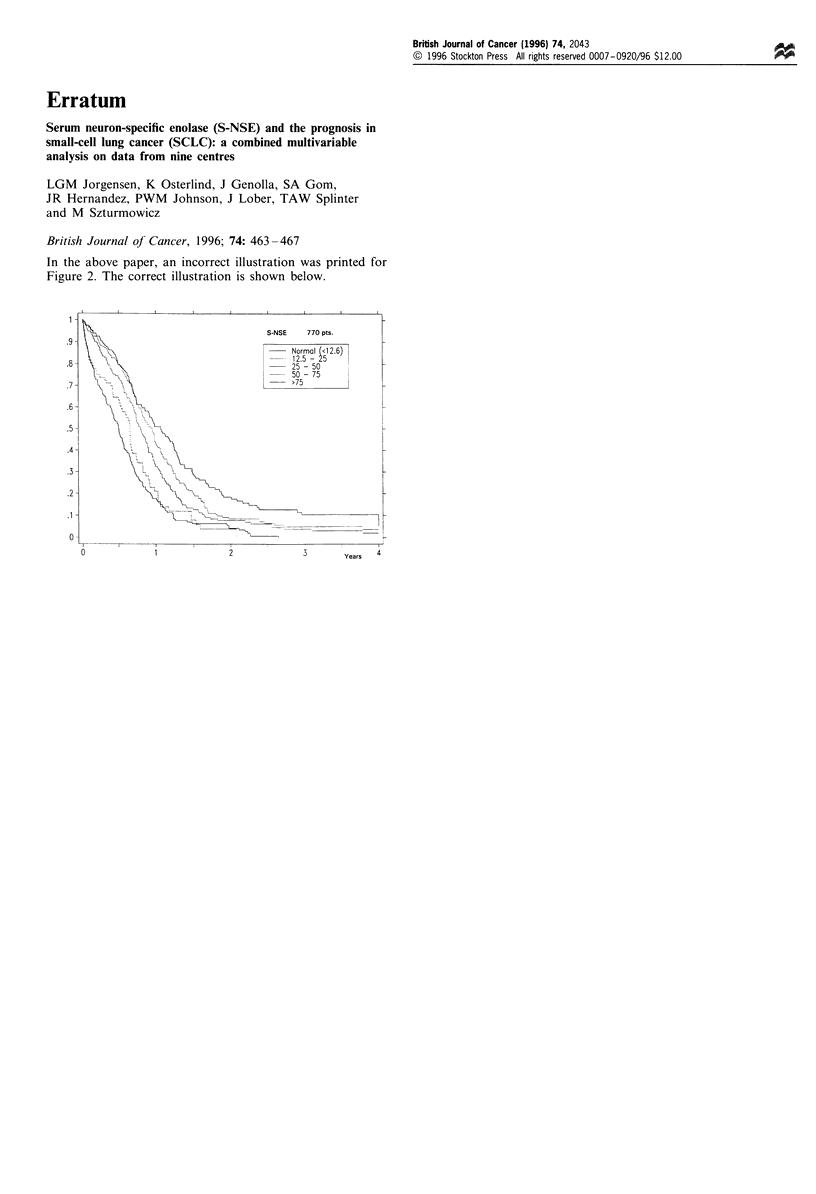# Serum neuron-specific enolase (S-NSE) and the prognosis in small-cell lung cancer (SCLC): a combined multivariable analysis on data from nine centres

**Published:** 1996-12

**Authors:** 


British Journal of Cancer (1996) 74, 2043
© 1996 Stockton Press All rights reserved 0007-0920/96 $12.00

### Erratum

**Serum neuron-specific enolase (S-NSE) and the prognosis in small-cell lung cancer (SCLC): a combined multivariable analysis on data from nine centres**

LGM Jorgensen, K Osterlind, J Genolla, SA Gom, JR Hernandez, PWM Johnson, J Lober, TAW Splinter and M Szturmowicz

*British Journal of Cancer*, 1996; **74:** 463–467

In the above paper, an incorrect illustration was printed for Figure 2. The correct illustration is shown below.